# The linkage of depressive and anxiety disorders with the expected labor market affiliation (ELMA): a longitudinal multi-state study of Danish employees

**DOI:** 10.1007/s00420-022-01906-z

**Published:** 2022-07-20

**Authors:** Jacob Pedersen, Elisabeth Framke, Sannie Vester Thorsen, Kathrine Sørensen, Malene Friis Andersen, Reiner Rugulies, Svetlana Solovieva

**Affiliations:** 1 National Research Centre for the Working Environment, Copenhagen, Denmark; 2 The Danish Multiple Sclerosis Registry, Copenhagen University Hospital, Copenhagen, Denmark; 3 Finnish Institute of Occupational Health, Helsinki, Finland

**Keywords:** Multi-state, Work, Sickness absence, Unemployment, Mental disorders

## Abstract

**Objective:**

Depressive and anxiety disorders are prevalent among employees in general. Still, knowledge regarding the contribution of these disorders to the dynamics of the labor market in terms of working time, sickness absence, and unemployment is scarce. We aim to quantify the linkage of depressive and anxiety disorders with labor market participation using the expected labor market affiliation method (ELMA), in a large sample of Danish employees.

**Methods:**

We combined three survey waves on occupational health with six high-quality national registers in *N* = 43,148 Danish employees, of which the 2012 survey contributed 29,665 person years, the 2014 survey 33,043 person years, and the 2016 survey 35,375 person years. We used the new ELMA method to estimate the multi-state transition probabilities and 2-year expected time in work, sickness absence, and unemployment. Depressive and anxiety disorders were assessed by the Major Depression Inventory and the SCL-ANX4 scales, respectively. We adjusted for multiple variables by applying inverse probability weighting in groups of gender and age.

**Results:**

Depressive and anxiety disorders among employees link to reduced labor market affiliation by significantly changed transitions probabilities between the labor markets states, viewed as reduced working time by 4–51 days (in two years), increased time in sickness absence by 6–44 days (in two years), and unemployment by 6–12 days (in two years) when compared to employees without depression or anxiety disorders. The results were most pronounced for women employees and for employees with both depression and anxiety disorders.

**Conclusions:**

The study reveals detailed insight into what extent depression and anxiety disorders influence the labor market affiliation, in terms of the complex interrelation between working time, sickness absence, and unemployment. The study emphasizes the importance of preventing and handling depressive and anxiety disorders among employees for strengthening work participation.

**Supplementary Information:**

The online version contains supplementary material available at 10.1007/s00420-022-01906-z.

## Introduction

Depressive and anxiety disorders were the two most common mental disorders in the European region in 2015, with 44.3 million and 37.3 million individuals, respectively, being affected (World Health Organization–Regional Office for Europe 2019), and among the top ten leading causes of disability (Vos et al. 2016). In Denmark, mentally ill health is increasing. The Danish health authorities have measured an increase in the mental illness from 2010 until the latest measurement in 2021, in particularly among the young and the women (Danskernessundhed.dk 2021). In 2015, anxiety was the third most frequent among new cases of illness, next to diabetes and ischemic heart disease. Depressive disorders caused 703 DALY’s per 100.000 individuals and anxiety disorders for 481 DALY’s, hence, the disease burden of the disorders in Denmark is similar to other high-income countries (Sundhedsstyrelsen 2022).

There is evidence of high co-occurrence of depression and anxiety, with approximately half of adults diagnosed with a depressive or anxiety disorder exhibiting these disorders simultaneously (Lamers et al. 2011). However, knowledge of the comorbid effect of these two prevalent mental disorders on labor market affiliation is sparse. Until now, it has been shown that depression and anxiety among workers increase the risk of sickness absence from work (Virtanen et al. 2011) and the risk of recurrent sickness absence (Knudsen et al. 2013) and imply increased time in sickness absence (Koopmans et al. 2010). In a large Finish study, it has been shown that early onset of depressive and also anxiety disorders (individuals between age 15 and 25 years) significantly decreases the likelihood of having secondary or higher education, decreases the work-life course likelihood of being employed, and increases the risk of having a total income below the median earning level (Hakulinen et al. 2019).

Multi-state modeling has proven to be an important tool for better understanding the dynamics of the labor market (Lie et al. 2017; Pedersen et al. 2020; Robroek et al. 2020) including studies of mental diseases in terms of depression (Pedersen et al. 2019). The expected Labor Market Affiliation method (ELMA), recently developed by Pedersen (Pedersen et al. 2021), relies on multi-state modeling of the labor market system, by the estimation of transitions probabilities and the expected state durations. In addition, the ELMA method provides the means to include time-dependent variables, weights, and multilevel adjustment.

The aim of the study is to quantify the impact of depressive and anxiety disorders on labor market participation for a large sample of Danish employees. We do this utilizing the ELMA method in a prospective study to analyze the transitions between multiple labor market outcomes among employees with and without depressive and anxiety disorders.

## Methods

### Study design and source population

This longitudinal study uses a linkage of registers and survey data on depressive and anxiety disorders from three successive waves of the Danish Work Environment and Health questionnaire (WEHD) conducted in the years 2012, 2014, and 2016 (Johnsen et al. 2019; Thorsen et al. 2019). The linkage was conducted by an encrypted version of the central person register number (CPR-Administration 2021). All WEHD responders, aged 18–64 years, were included and followed in registers for two years from the day they filled in the questionnaire.

The WEHD surveys were linked with the following registers, provided by Statistics Denmark: (1) Danish Labor Market Accountant (LMA) register, (2) Register of Work Absences (RoWA), (3). Education register, (4) Emigration and Immigration register and (5) Death register. The LMA register contains information on all major social benefits payments, including unemployment, sickness absence, disability pension, pension, and all salary payments reported to the tax authorities from 2008 onwards.

The RoWA register is a linkage of the Absence and Employment -register (FRAN) and the Periods of Absence -register (FRPE), both from Statistics Denmark. FRPE includes information about sickness absence spells already from the first day of absence and FRAN includes date-based employment information of both the employees with and without sickness absence spells (Thorsen et al. 2019). The RoWA register contains records of both public and private employees. The date-based records of sickness absence spells are complete for all public employees and for all large private companies with more than 250 employees. The RoWA contains a yearly weighted sample of middle-sized companies with 10–250 employees. This means that RoWA is covering approximately 37% of all private employees in Denmark (Thorsen et al. 2019). The RoWA does not include small private companies with less than 10 employees. Small companies represent a large part of private companies (approx. 260,000 small private companies exist in Denmark) and they are not represented in our study (Smvdanmark.dk 2018). The RoWA register contains weights for making the private sample representative to all private employees in companies with 10 employees or more. The Education register contains records of the highest education level completion for all Danes. The Emigration and Immigration register contains dates on all emigrations and immigrations in Denmark. The Death-register includes dates for all deceased Danes.

The linkage contains individual and date-based information on labor market affiliation and individual characteristics retained from the surveys etc.

### Study sample and data preparation

The WEHD data included 67 053 individuals of which 63 912 (95.3%) individuals were eligible for the current study. Receivers of disability pension or retires at the start of the follow-up period (*n* = 2 945), individuals older than 64 years at the start of the follow-up (*n* = 195, 28% women), and not found in the LMA register (*n* = 1) were excluded.

In the RoWA register (i) all records for public employment has the weight one, and (ii) all records for private employment have a specialized weight that is constructed by Statistics Denmark based on the sampling probability according to the yearly weighted sample of companies. RoWA only include records of individuals in employment, but in this study the weights were carried forward in the LMA register, to include periods of unemployment, etc., but only until a new employment period.

Records from the LMA register that could not be linked to private or public employment in the RoWA register were deleted (~ 7%. 0.6 million records). Similarly, records of private employments without a weight (9%), and public employment period with a specialized weight (0.1%) were deleted. Moreover, records with missing answers on questions regarding depression or anxiety were deleted (*N* = 3021).

Since an individual may have attended one, two, or three survey waves (hence 2012, 2014, and 2016)—multiple follow-up periods are possible. The final sample contains 59,540 follow-up periods—of the *N* = 43,148 individuals, 77% participated only in one survey, 8% participated in two of the three survey waves, and 15% participated in all three survey waves.

For the analyses, the study sample was divided into four subsamples according to age and gender. The two age groups were 18–47 years and 48–64 years, the division was at the median age. The division into only two groups was to secure a sufficient number of individuals in each subgroup.

### Depressive and anxiety disorders

Depressive disorders were measured with the 12-item Major Depression Inventory (MDI), with the sum score ranging between 0 and 50 (please see supplementary material A) (Bech et al. 2001; Olsen et al. 2003). The MDI has been validated in both the general population and among patients in the clinical setting (Bech et al. 2001; Cuijpers et al. 2007). In accordance with a clinical validation study by Bech (Bech et al. 2015), we categorized study participants as having a depressive disorder, if they scored ≥ 21 on the MDI-scale, indicating a mild to severe level of depression.

Anxiety was measured by the SCL-ANX4 scale containing four questions. In accordance with a clinical validation study by Christensen (Christensen et al. 2005), we categorized study participants with an anxiety disorder, if at least three out of the four dichotomized anxiety symptoms were present on the SCL-ANX4 scales.

Next, we categorized the respondents into four groups: (1) Neither depressive nor anxiety disorders, (2) Depressive disorder without anxiety disorder, (3) Anxiety disorder without the depressive disorder, 4) Both depressive and anxiety disorders. The questions regarding the MDI scale and the SCL-ANX4 scale are presented in supplementary material A.

### Covariates and weights

The analyses included seven covariates that have been used in previous studies on mental health and labor market affiliation in relation to self-perceived stress, life course analysis of depression symptoms, and psychiatric work disability (Thorsen et al. 2019; Pedersen et al. 2019, 2021; Virtanen et al. 2011). The covariates are associated with adverse health outcomes, possible through selection, e.g. selection into part-time work, or through causation, e.g. smoking and sickness absence.

Four of the variables were taken from the WEHD survey data: (1) working time arrangement (part-time: < 37 h per week; full time: ≥ 37 h per week), (2) body mass index (BMI, kg/m^2^) (underweight: BMI < 18.0; normal weight: 18.5 ≤ BMI < 25.0; overweight: 25.0 ≤ BMI < 29.9; and obese: BMI ≥ 29.9), (3) smoking (current smoker vs. former or never smoker), and (4) disease treatment—in terms of a dichotomous variable indicating whether the individual had been treated for one of the following diseases (no/yes): asthma, diabetes, atherosclerosis or blood clot in the heart, blood clot in the brain (cerebral hemorrhage), cancer, back diseases, migraine, or other long-term diseases. One variable was obtained from the FRAN register: employment sector (private/public), and one variable were obtained from the education registers: the highest accomplished education (low/middle/high). The last variable “number of survey waves” was constructed to account for the number of WEHD survey waves the individual had attended – “1 of 3”, “2 of 3”, and “3 of 3”. All covariates were determined at baseline but the level of education and employment sector was moreover allowed to change during the follow-up period.

### Labor market affiliation

**Fig. 1:**
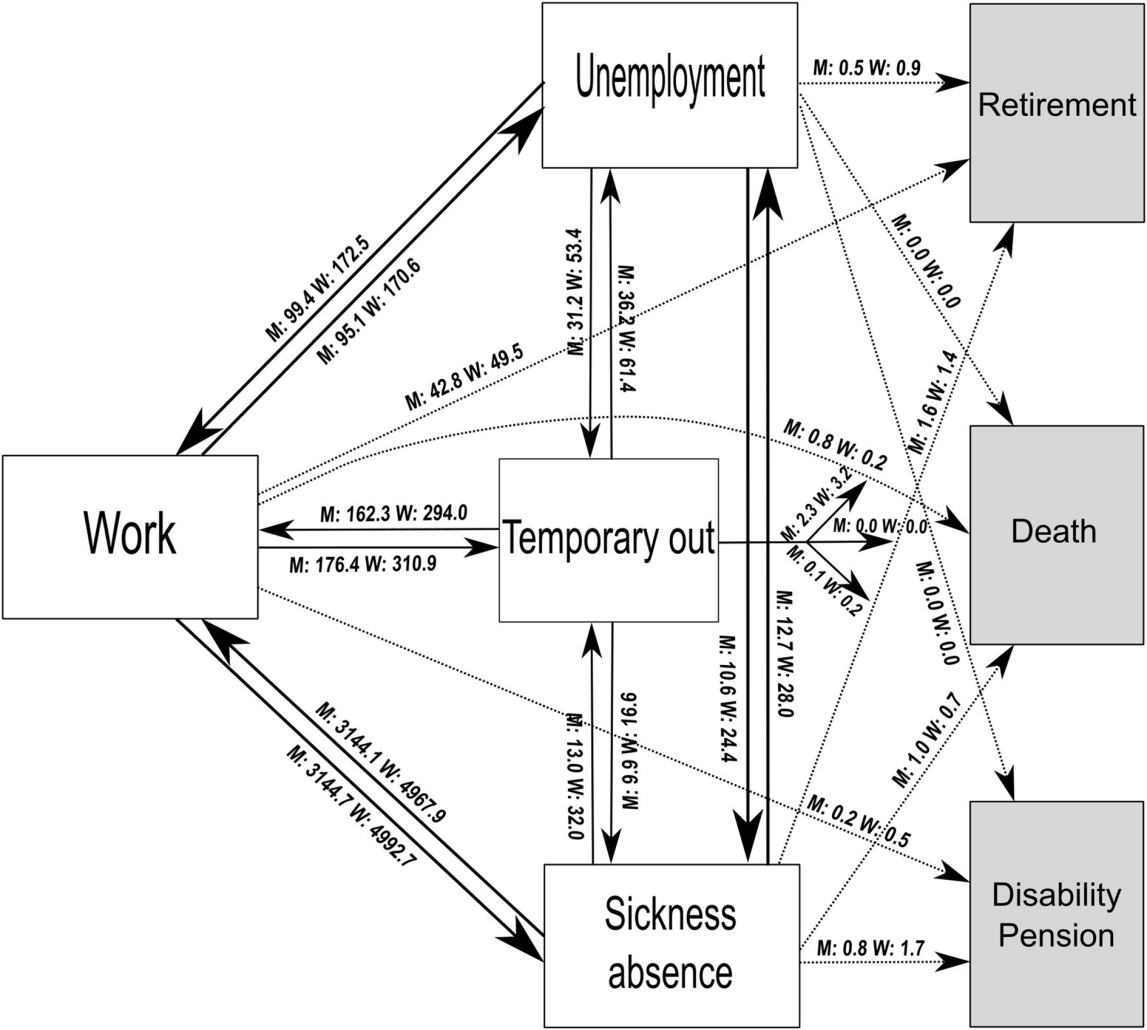
The multi-state model including the prevalence of each transition per 1000 individuals, for men (*M*) and for women (*W*). Transitions as arrows, recurrent states as white boxes, and absorbing states as gray boxes. Example—during the follow-up period men experienced approx. 3,144,700 transitions from work to sickness absence

The labor market affiliation was modeled by seven mutually exclusive labor market states based on the longitudinal registrations of the LMA and the RoWA registers, illustrated by boxes in Fig. 1. Of the seven states, four were categorized as recurrent states- meaning that individuals may enter and leave this state multiple times—transitions are illustrated by arrows in Fig. 1: (1) Work, reflecting the periods of receiving salary payments and not simultaneously registered as sick-listed. (2) Sickness absence, for periods when the individual is registered as sick-listed by the employer and for periods of sickness absence benefit payments. (3) Unemployment, for periods, when a person received any type of social benefit related to unemployment, given the condition that the person is immediately available for work if such opportunity arises. (4) A temporarily out state, for periods when an individual was not in the work, sickness absence, or unemployment states but had the possibility of returning to those states. This state contained, for example, periods of maternity leave, emigration, education, and periods with no registration. The three remaining states were all-absorbing states, meaning that no further transitions were possible after the first entry to the state: (5) disability pension when receiving disability retirement pension due to personal disability. (6) Retirement with age retirement pension or the voluntary retirement pension. (7) Death (supplementary material B, contains a short introduction to the Danish labor market and social system).

### Statistical analysis

We used the ELMA method developed by Pedersen (Pedersen et al. 2021), for analyzing the transition probabilities between the states of the multi-state model and for estimating the expected state durations during follow-up. The ELMA incorporates both time-dependent variables and time-dependent weights in terms of e.g. inverse probability weights. SAS 9.4 software was used for the ELMA analysis including the procedure PHREG and otherwise custom-made code.

For each subsample of gender and age groups, we estimated the time-dependent baseline probability for every transition of the multi-state model according to the reference value of the covariates. The transitions probabilities for the non-reference values were estimated by adjusting the corresponding baseline probabilities with estimates derived from Cox-proportional hazard regression. The Cox-regressions were conducted on the entire multi-state model with the data arranged in a long format (de Wreede et al. 2010). We tested the proportional hazard assumption of the cox-regression by visual inspection of the transition probability curves and found them satisfactory. Based on the transition probabilities we estimated the state probabilities and then the state durations – from day one and until day 730 (two years). An analysis of variance was then conducted on five hundred re-samples, assuming normally distributed state duration.

To increase the strength of the area estimation, all variables except for the combined depressive and anxiety disorder variable, were incorporated into the model as inverse probability weights. A combined weight was incorporated into the Cox regression and the area estimation, as a multiplication of the weights from the employment register and the inverse probability weight. For light comparison with and control of the ELMA results, a crude estimate of the time spent in each state was made. This was done by summing the time spent in each state during the follow-up period and then dividing it by the number of individual follow-up periods.

## Results

Table 1 shows that—the study sample consisted of 25,392 women (59%) and 17,756 men (41%). Combined depressive and anxiety disorders were more frequently reported by women than men. Depression without anxiety was the most frequent among young women.

**Table 1:** Descriptive baseline characteristics at the start of the first follow-up period

Age group (years)		Men		Women	
		18–47	48–64	18–47	48–64
Total—*n* (mean age)		8971 (37.2)	8785 (55.6)	13,494 (37.0)	11,898 (55.2)
		*N* (%)	*N* (%)	*N* (%)	*N* (%)
Depressive/anxiety					
No/No		6537 (72.9)	6767 (77.0)	8789 (65.1)	8378 (70.4)
No/Yes		283 (3.2)	347 (3.9)	609 (4.5)	547 (4.6)
Yes/No		1472 (16.4)	1002 (11.4)	2633 (19.5)	1780 (15.0)
Yes/Yes		679 (7.6)	669 (7.6)	1463 (10.8)	1193 (10.0)
Working time					
Full-time		7907 (88.1)	8050 (91.6)	8640 (64.0)	7558 (63.5)
Part-time		942 (10.5)	580 (6.6)	4686 (34.7)	4118 (34.6)
Not available		122 (1.4)	155 (1.8)	168 (1.2)	222 (1.9)
Body mass index					
Underweight		40 (0.4)	19 (0.2)	312 (2.3)	183 (1.5)
Normal		4192 (46.7)	3118 (35.5)	8044 (59.6)	6346 (53.3)
Overweight		3456 (38.5)	4167 (47.4)	3184 (23.6)	3537 (29.7)
Obesity		1189 (13.3)	1423 (16.2)	1655 (12.3)	1674 (14.1)
Not available		94 (1)	58 (0.7)	299 (2.2)	158 (1.3)
Smoking					
Non-smoker		7036 (78.4)	6959 (79.2)	10,982 (81.4)	9536 (80.1)
Smoker		1927 (21.5)	1823 (20.8)	2503 (18.5)	2340 (19.7)
Not available		8 (0.1)	3 (0)	9 (0.1)	22 (0.2)
Disease treatment					
No		7152 (79.7)	6072 (69.1)	9720 (72.0)	7890 (66.3)
Yes		1819 (20.3)	2713 (30.9)	3774 (28.0)	4008 (33.7)
Employment sector					
Private		5588 (62.3)	5218 (59.4)	4051 (30.0)	2876 (24.2)
Public		3383 (37.7)	3567 (40.6)	9443 (70.0)	9022 (75.8)
Highest educational level					
Short		979 (10.9)	1395 (15.9)	895 (6.6)	1570 (13.2)
Medium		3662 (40.8)	3972 (45.2)	4876 (36.1)	4731 (39.8)
Long		4294 (47.9)	3355 (38.2)	7691 (57.0)	5561 (46.7)
Not available		36 (0.4)	63 (0.7)	32 (0.2)	36 (0.3)
Number of survey waves					
1 of 3		7036 (78.4)	6681 (76.1)	10,416 (77.2)	9002 (75.7)
2 of 3		816 (9.1)	619 (7.0)	1360 (10.1)	839 (7.1)
3 of 3		1119 (12.5)	1485 (16.9)	1718 (12.7)	2057 (17.3)

Figure 1 shows that the transitions between the work and the sickness absence states were the most frequent during follow-up, with the highest prevalence being for women. Transitions between work and unemployment were less frequent than the transitions between work and the temporarily out state. Moreover, the transitions between work and temporarily out states were most prevalent among women. The prevalences of transitions to the absorbing states were generally very low.

Table 2 show that—during the two-year follow-up—working individuals with both depressive and anxiety disorders had the highest hazard ratios. Few exceptions were seen—for 18–47 years old working women and 48–64 years old working men. Working 18–47 years old women with anxiety and no depressive disorders had a higher risk of being unemployed than similar-aged women with both depressive and anxiety disorders, but with overlapping confidence intervals (HR 1.91 for anxiety and 1.64 for both disorders). The risk of sickness absence was somewhat similar among 18–47 years old women with depressive disorders alone or in combination with anxiety (HR 1.33 and 1.31, respectively). Working women with depressive disorders alone or in combination with anxiety had a higher risk of unemployment than working women reporting solely anxiety disorders (HR 1.28 for anxiety, 1.60 for depression, and 2.18 for both disorders). The reference group are similarly aged employees of the same gender without depression or anxiety disorders.

**Table 2:** Hazard ratio and 95% confidence interval for transitions between the three main states: work, sickness absence, and unemployment, by gender and age-group

Transition	Depressive/Anxiety	Men		Women	
		Aged 18–47 years	Aged 48–64 years	Aged 18–47 years	Aged 48–64 years
		HR (95% CI)	HR (95% CI)	HR (95% CI)	HR (95% CI)
Work to Sickness absence					
Dep. No/Anx. No (ref.)		1.00 (–)	1.00 (–)	1.00 (–)	1.00 (–)
Dep. No/Anx. Yes		1.12 (0.97–1.29)	1.39 (1.15–1.68)^a^	1.20 (1.10–1.32)^a^	1.12 (1.02–1.24)^a^
Dep. Yes/Anx. No		1.25 (1.11–1.40)^a^	1.35 (1.21–1.50)^a^	1.33 (1.25–1.42)^a^	1.30 (1.22–1.38)^a^
Dep. Yes/Anx. Yes		1.33 (1.16–1.54)^a^	1.53 (1.30–1.81)^a^	1.31 (1.18–1.46)^a^	1.49 (1.34–1.66)^a^
Work to unemployment					
Dep. No/Anx. No (ref.)		1.00 (–)	1.00 (–)	1.00 (–)	1.00 (–)
Dep. No/Anx. Yes		1.54 (0.61–3.86)	1.63 (0.77–3.44)	1.91 (1.05–3.45)^a^	1.28 (0.72–2.28)
Dep. Yes/Anx. No		1.15 (0.62–2.11)	1.63 (0.96–2.76)	1.32 (0.93–1.87)	1.60 (1.09–2.34)^a^
Dep. Yes/Anx. Yes		1.89 (1.03–3.46)^a^	1.39 (0.81–2.37)	1.64 (1.13–2.39)^a^	2.18 (1.50–3.15)^a^
Sickness absence to work					
Dep. No/Anx. No (ref.)		1.00 (–)	1.00 (–)	1.00 (–)	1.00 (–)
Dep. No/Anx. Yes		0.77 (0.54–1.10)	0.92 (0.58–1.48)	0.85 (0.59–1.23)	0.87 (0.69–1.11)
Dep. Yes/Anx. No		0.73 (0.60–0.90)^a^	0.92 (0.69–1.22)	0.77 (0.64–0.92)^a^	0.60 (0.48–0.75)^a^
Dep. Yes/Anx. Yes		0.45 (0.32–0.65)^a^	0.64 (0.48–0.86)^a^	0.56 (0.46–0.68)^a^	0.52 (0.44–0.61)^a^
Sickness absence to unemployment					
Dep. No/Anx. No (ref.)		1.00 (–)	1.00 (–)	1.00 (–)	1.00 (–)
Dep. No/Anx. Yes		0.60 (0.09–3.90)	1.66 (0.33–8.42)	1.83 (0.92–3.66)	1.43 (0.68–3.03)
Dep. Yes/Anx. No		0.98 (0.46–2.11)	1.53 (0.56–4.13)	1.91 (1.22–2.99)^a^	1.49 (0.91–2.44)
Dep. Yes/Anx. Yes		1.07 (0.46–2.49)	2.34 (1.11–4.94)^a^	3.30 (1.86–5.84)^a^	2.46 (1.11–5.47)^a^
Unemployment to work					
Dep. No/Anx. No (ref.)		1.00 (–)	1.00 (–)	1.00 (–)	1.00 (–)
Dep. No/Anx. Yes		0.47 (0.30–0.75)^a^	0.98 (0.56–1.69)	1.18 (0.67–2.08)	0.85 (0.42–1.73)
Dep. Yes/Anx. No		0.72 (0.45–1.14)	0.45 (0.25–0.81)^a^	0.62 (0.46–0.82)^a^	0.69 (0.47–1.02)
Dep. Yes/Anx. Yes		0.75 (0.47–1.22)	0.42 (0.23–0.76)^a^	0.58 (0.43–0.79)^a^	0.67 (0.43–1.03)
Unemployment to sickness absence					
Dep. No/Anx. No (ref.)		1.00 (–)	1.00 (–)	1.00 (–)	1.00 (–)
Dep. No/Anx. Yes		0.76 (0.17–3.47)	0.14 (0.02–1.25)	1.19 (0.62–2.27)	1.51 (0.86–2.65)
Dep. Yes/Anx. No		0.44 (0.16–1.23)	0.44 (0.12–1.60)	1.11 (0.67–1.83)	1.26 (0.76–2.08)
Dep. Yes/Anx. Yes		1.51 (0.73–3.13)	0.74 (0.35–1.56)	1.87 (1.03–3.40)^a^	1.87 (1.10–3.20)^a^

*Dep.* Depressive, *Anx.* Anxiety, *HR* Hazard Ratio, *CI* Confidence Interval, *Ref.* Reference

^a^5% Significant

**Fig. 2:**
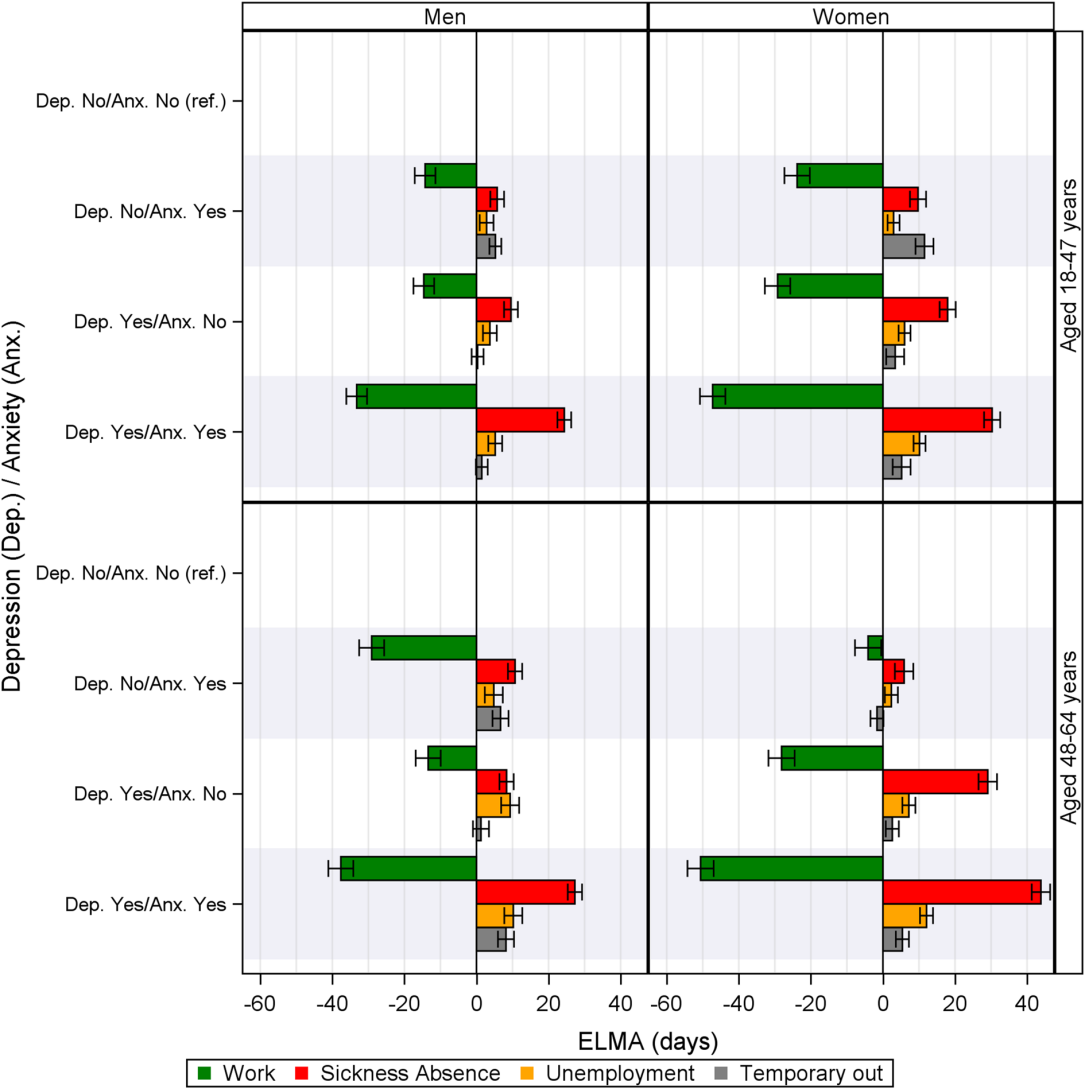
The ELMA results by the expected duration (±) in two year of working time, sickness absence, unemployment, and temporarily out (days) when compared to the absolute duration time of individuals without depression and anxiety. Grouped by gender, age, and the combination of disorders

Table 3 shows that—during the two-year follow-up—men without depressive and anxiety disorders were expected to have 694 (aged 18–47 years) and 670 (aged 48–64 years) working days, respectively (Table 3). The corresponding value for working time expectancy among women was 658 (aged 18–47 years) and 660 (aged 48–64 years) working days. Figure 2 shows that the expected working time was significantly decreased for all groups with depressive and/or anxiety disorders, except for women with solely anxiety disorders aged 48–64 years. For men aged 48–64 years, a lesser decrease in working time was observed for individuals with depressive disorders, than those with anxiety disorders and those with both disorders. The number of sickness absence days and unemployment days were higher for individuals with either depressive or anxiety disorders, or with both disorders, and days in work were fewer. Individuals with both disorders experience the highest number of sickness and unemployment days and there was a tendency for depressive disorders to be associated with more sickness and unemployment days than anxiety disorders. The reference group are similarly aged employees of the same gender without depression or anxiety disorders.

**Table 3:** The ELMA and Crude mean results (in days incl. 95% confidence interval) of the expected change ( ±) in two year by the duration of working time, sickness absence, unemployment, and temporarily out when compared to the absolute duration time of individuals without depressive and anxiety disorders (reference). Grouped by gender, age, and by disorder: anxiety, depressive, or both. Supplementary tables (A), shows the additional results of the three absorbing states

Gender	Age	Depressive/anxiety		Work		Sickness absence		Unemployment		Temporary out	
				ELMA	Crude	ELMA	Crude	ELMA	Crude	ELMA	Crude
				Days (95% CI)	Days	Days (95% CI)	Days	Days (95% CI)	Days	Days (95% CI)	Days
Men	18–47 years	Dep. No/Anx. No (ref.)		694.4 (692.3:696.4)^a^	582.3	13.3 (11.9:14.6)^a^	10.1	4.3 (2.9:5.6)^a^	5.1	14.5 (13.3:15.7)^a^	14.7
		Dep. No/Anx. Yes		– 14.3 ( – 17.1: – 11.4)^a^	+ 7.6	+ 5.7 (3.8:7.7)^a^	+ 3.5	+ 2.8 (0.9:4.7)^a^	+ 2.2	+ 5.2 (3.6:6.9)^a^	+ 5.8
		Dep. Yes/Anx. No		– 14.6 ( – 17.5: – 11.7)^a^	– 28.6	+ 9.6 (7.7:11.5)^a^	+ 9.5	+ 3.7 (1.8:5.6)^a^	+ 3.2	+ 0.3 ( – 1.3:2.0)	+ 4.1
		Dep. Yes/Anx. Yes		– 33.2 ( – 36.1: – 30.4)^a^	– 42.0	+ 24.4 (22.5:26.3)^a^	+ 17.9	+ 5.2 (3.3:7.1)^a^	+ 9.9	+ 1.5 ( – 0.2:3.1)	+ 5.5
	48–64 years	Dep. No/Anx. No (ref.)		670.3 (667.9:672.8)^a^	578.9	19.6 (18.2:21.0)^a^	15.7	3.2 (1.4:5.0)^a^	4.1	3.6 (2.0:5.2)^a^	4.4
		Dep. No/Anx. Yes		– 29.1 ( – 32.6: – 25.6)^a^	– 8.3	+ 10.7 (8.7:12.7)^a^	+ 6.0	+ 4.8 (2.3:7.3)^a^	+ 4.3	+ 6.7 (4.4:8.9)^a^	+ 2.5
		Dep. Yes/Anx. No		– 13.4 ( – 16.9: – 9.9)^a^	– 13.0	+ 8.3 (6.4:10.3)^a^	+ 12.3	+ 9.3 (6.8:11.8)^a^	+ 4.2	+ 1.2 ( – 1.0:3.5)	+ 3.6
		Dep. Yes/Anx. Yes		– 37.6 ( – 41.1: – 34.2)^a^	– 40.5	+ 27.3 (25.3:29.3)^a^	+ 26.7	+ 10.2 (7.7:12.7)^a^	+ 0.4	+ 8.2 (6.0:10.4)^a^	+ 10.2
Women	18–47 years	Dep. No/Anx. No (ref.)		657.7 (655.2:660.2)^a^	611	26.6 (25.0:28.2)^a^	23.2	6.2 (5.1:7.4)^a^	6.2	36.9 (35.2:38.7)^a^	35.8
		Dep. No/Anx. Yes		– 23.8 ( – 27.3: – 20.3)^a^	– 18.6	+ 9.7 (7.5:12.0)^a^	+ 6.8	+ 3.0 (1.3:4.6)^a^	+ 4.6	+ 11.5 (9.0:14.0)^a^	+ 9.6
		Dep. Yes/Anx. No		– 29.2 ( – 32.8: – 25.7)^a^	– 35.1	+ 18.0 (15.7:20.2)^a^	+ 16.9	+ 6.0 (4.3:7.7)^a^	+ 6.4	+ 3.4 (0.9:5.9)^a^	+ 4.0
		Dep. Yes/Anx. Yes		– 47.2 ( – 50.8: – 43.7)^a^	– 63.3	+ 30.3 (28.1:32.5)^a^	+ 34.7	+ 10.2 (8.5:11.8)^a^	+ 11.7	+ 5.2 (2.7:7.7)^a^	+ 6.1
	48–64 years	Dep. No/Anx. No (ref.)		660.0 (657.5:662.6)^a^	625.3	29.3 (27.5:31.1)^a^	25.2	5.4 (4.1:6.7)^a^	5.3	3.3 (2.0:4.6)^a^	4.2
		Dep. No/Anx. Yes		– 4.1 ( – 7.8: – 0.5)	– 17.3	+ 5.9 (3.3:8.4)^a^	+ 5.8	+ 2.3 (0.5:4.1)	+ 2.2	– 1.6 ( – 3.5:0.2)	– 2.6
		Dep. Yes/Anx. No		– 28.1 ( – 31.8: – 24.5)^a^	– 32.5	+ 29.1 (26.5:31.6)^a^	+ 24.0	+ 7.2 (5.4:9.0)^a^	+ 7.5	+ 2.6 (0.8:4.4)^a^	+ 1.7
		Dep. Yes/Anx. Yes		– 50.6 ( – 54.2: – 47.0)^a^	– 63.8	+ 43.8 (41.3:46.4)^a^	+ 42.3	+ 12.1 (10.3:13.9)^a^	+ 10.9	+ 5.4 (3.6:7.2)^a^	+ 4.7

*Dep.* Depressive, *Anx.* Anxiety, *ELMA* Expected Labor Market Affiliation

^a^5% significant

During the two-year follow-up, the expected working time of men with depressive and anxiety disorders was reduced by 33 and 38 days for the younger and older age group, respectively. Reduction in the expected working time among older men experiencing anxiety disorders without depressive disorders was larger than among younger men with the same disorders (by 29 and 14 days for the aged 48–64 and 18–47 years, respectively). The expected time of early retirement (supplementary table C) was + 32 days for women and + 27 days for men – when compared with similar-aged employees of the same gender without depression or anxiety disorders.

## Discussion

In this longitudinal study, we examined a two-year labor market participation of employees after reporting depressive and/or anxiety disorders. By using the novel ELMA method on three waves of the WEHD survey linked to six national registers, we found that working individuals with depressive and anxiety disorders had a higher risk of sickness absence and unemployment. Moreover, when sickness absent, they had a higher risk of being unemployed, and when unemployed or sickness absent, they had less chance of returning to work. Overall, employees with either depressive or anxiety disorders had during the next two years less working time, more sickness absence time, and more unemployment time, compared to individuals without depressive and anxiety disorders.

The decrease in working time and increase in sickness absence and unemployment times were more pronounced among employees reporting a combination of depressive and anxiety disorders than employees having only one type of the disorders. Among those with both types of disorders, women had a larger working time decrease and a larger increase in sickness absence and unemployment than men. In the group with solely depressive disorders, and no anxiety disorders, women compared to men had twice the decline in working time and approximately twice the increase of sickness absence time. In the group with solely anxiety disorders, women in the young age group had a higher increase/decrease than men, but in the old age group men had higher increase/decrease than women.

Young men and women with either depressive or anxiety disorders had comparable changes in the labor market affiliation. In the oldest age group, the change in labor affiliation appeared to be more pronounced among men with anxiety disorders than men with depressive disorders and likewise, depressive disorders appeared to relate to more lost working time and increased sickness absence, etc. than anxiety disorders among women.

The crude mean tends to underestimate the reference level of working time and time of sickness absence when compared to the ELMA estimate. But for confirmation of the results, the ELMA and crude estimates generally point in the same direction.

### Comparison with previous studies

Our study is the first to apply the ELMA multi-state approach to explore the linkage of depressive and anxiety disorders with the labor market affiliation. Earlier studies have usually focused on depression and either analyzed only single transitions e.g. from work to long-term sickness absence or disability pension (Hjarsbech et al. 2011; Holma et al. 2012; Thorsen et al. 2013), focused on recurrent sickness absence (Knudsen et al. 2013), or taken a life course perspective to quantify the effect on working life expectancy (Pedersen et al. 2019; Hakulinen et al. 2019). Other studies focus on the economic consequences and find significantly decreased income levels and production loss among workers having depressive symptoms or disorders—with additionally subsequent risk of unemployment (Stewart et al. 2003; Whooley et al. 2002).

Like previous studies, we found that depressive and/or anxiety disorders were associated with an increased risk of sickness absence among working men and women, and a decreased likelihood of returning to work (Knudsen et al. 2013; Virtanen et al. 2011). In line with the study by Andreeva (Andreeva et al. 2015), we found that depression increases the likelihood of transitioning from work to unemployment among women. Moreover, we found that young men and women in both age groups—with anxiety and depressive disorders—had an increased risk of unemployment. In contrast to Jefferis (Jefferis et al. 2011) our results were statistically significant even when adjusting for education level and employment sector.

The present study found reduced working and increased sickness absence and unemployment time for individuals having depressive disorders during a 2-year follow-up. The result are in line with the life course study by Pedersen (Pedersen et al. 2019) on the impact of depression on working life expectancy and working-years loss, and (Banerjee et al. 2017) in terms of estimated absenteeism from work for employees with mental illness.

The results suggest a prevention potential in relation to reducing the loss of working time for employees reporting depressive and anxiety disorders by lowering the risk of sickness absence. However, there exists only a spared number of intervention studies with a positive proven effect (Henderson et al. 2011; Nexø et al. 2018). One of such studies contains a method by which employers screen the employees for mental health disorders, to decrease the symptoms, gain higher job retention, and gain more hours working (Wang et al. 2007). The results additionally indicate a potential for reducing the time in sickness absence, by increasing the likelihood of returning to work – especially for sick-listed employees having both depression and anxiety disorders. Here stigmatization may be an obstacle for the sick-listed and the employer to overcome, as the sick-listed may find it difficult to seek help and the employer may have difficulties handling the return to the workplace if not familiar with the disorders of the employee (Gronholm et al. 2017).

### Strengths and limitations

The study strengths include a large study population of Danish employees from three survey waves, and the flexibility of the ELMA method made it possible to examine different aspects of the labor market affiliation—including adjustment for time-dependent variables and weights.

An additional strength is the use of all lengths of sickness absence. Most comparable register studies rely on registrations of long-term sickness absence benefits, which in Denmark concerns sickness absence of more than 30 continuous days.

The study includes limitations for consideration: (i) The sample represents a wide variety of Danish employees and the study is likely to be generalizable to the Danish workforce, and find use particular in countries with a comparable labor market system e.g. the Scandinavian countries. However, some caution should be taken on the WEHD, due to the lack of response from men, young employees, and people with many sickness absence days (Thorsen et al. 2018, 2019; Johnsen et al. 2019) and due to the limits of the RoWA register concerning small private companies (Smvdanmark.dk 2018). (ii) Only a few transitions to the disability pension and other pension states were observed during a 2-year follow-up, nevertheless, there is a small possibility of overestimating the time spent in those states, as these states in the model were absorbing. (iii) To secure sufficient statistical power throughout the analysis the study did not distinguish between full-time and part-time unemployment and sickness absence, though the Danish system contains both. Instead, all time in these states was treated only as full-time. This potentially overestimates the reduction in working time and similarly overestimates the time in sickness absence, as some of the time will be productive (part-time at work) and not full-time sickness absence. (iv) The use of survey data on depressive and anxiety disorders may cause non-response bias as individuals may find the depression and anxiety questions irrelevant or choose not to answer. (v) Additionally, the classification of variables, including disease by the survey data, does not allow for individual variable shifts during follow-up. This may cause misestimation, as, for example, the severity of the baseline level of depression and/or anxiety disorders may fluctuate during follow-up and possibly fade out. (vi) The study relates to the Danish social system and labor market system, which means that comparisons with other countries should be made with caution. However, the results may still make room for cross-country consideration on employees experiencing depressive and/or anxiety disorders. (vii) Moreover, it is likely that the results can be driven by additional causes not included in the study. For example, the study does not include information on the severity of the disorders, medication side effects, or person-related crises. Additionally, the use of the self-reported disorders instead of diagnosis-specific information suggests a risk of misclassification as the presence of each disorder has not been confirmed by a psychiatrist or doctor (viii) In addition, the study does not contain any information about previous mental states before baseline, which makes it difficult to determine and include a possible duration of depressive and/or anxiety disorders up to baseline.

## Conclusion

This study provides detailed new knowledge on the linkage between depressive and anxiety disorders with the labor market affiliation among Danish employees. Using the ELMA method we show that depressive and anxiety disorders are associated with noticeable loss of working time and increase of time in sickness absence and unemployment. The relationship was higher for employees with both disorders compared to employees with only one, it was higher for women compared to men, and it varied by age. The result of our study, i.e. how many lost workdays depression and anxiety causes, is an easily understandably number also for non-researchers. The loss in workdays, increase in sick days etc. highlight the importance of the prevention and handling of depression and anxiety disorders in the workplace and identifying employees with these disorders even though the cause may not be work-related. Further use of these numbers will, in future studies, be to calculate the cost of depression and anxiety for both the industry and society.

## Supplementary Information

Below is the link to the electronic supplementary material.

Supplementary file1 (PDF 930 KB)

## Data Availability

The SAS code can be shared upon reasonable request by authorized researchers after application to the NRCWE. Data is available on the Researcher access at Statistics Denmark, see www.dst.dk/en/TilSalg/Forskningsservice.
