# Supplementary material for: The linkage of depressive and anxiety disorders with the expected labor market affiliation (ELMA): a longitudinal multi-state study of Danish employees
Source: Int Arch Occup Environ Health. 2022 Jul 20;96(1):93–104. doi: 10.1007/s00420-022-01906-z (PMC9823083; doi:10.1007/s00420-022-01906-z)
Supplement: Supplementary file 1 — Supplementary file1 (PDF 930 KB) [file 420_2022_1906_MOESM1_ESM.pdf]

Supplementary material

A) - The depression and anxiety scales

The depression (MDI) scale contains twelve questions: "Have you felt low in spirits or sad?", "Have you lost interest in your daily activities?", "Have you felt lacking in energy and strength?", "Have you felt less self-confident?", "Have you had a bad conscience or feelings of guilt?", "Have you felt that life wasn't worth living?", "Have you had difficulty in concentrating, e.g., when reading the newspaper or watching television?", "Have you felt very restless?", "Have you felt subdued?", "Have you had trouble sleeping at night?", "Have you suffered from reduced appetite?", "Have you suffered from increased appetite?". All twelve questions are rated ranging from: "All the time", "Most of the time", "A lot of the time", "Some of the time", "A little of the time", "At no time". Please see (Bech et al. 2001) for scoring or the 'Major (ICD-10) Depression Inventory' added to the end of the supplementary material.

The anxiety (SCL-ANX4) scale contain four questions: "Feeling suddenly scared for no reason?", "Nervousness or shakiness inside?", "Spells of terror or panic?", and "You worry too much?" All four questions are rated on a Likert scale of distress ranging from: "Not at all", "A little", "Moderately", "Quite a bit" to "Extremely". The individual answers are dichotomized with "Not at all" as zero, and "A little" to "Extremely" as one.

B) - Jurisdictional context: the Danish labor market

The Danish labor market is characterized as a flexicurity system, with high labor market participation rates and low formal employment protection inflicting a high turnover of the workforce. In addition, the Danish system contains generous and accessible social benefits (e.g. sickness absence, unemployment, disability pension) (Madsen 2005).

A Danish employee will typically receive salary during a period of sickness absence, and if the sickness absence period exceeds 30 days, then the employer is entitled to compensation from the municipality for paying salary to the sick-listed employee in terms of sickness absence benefit. If an individual is unemployed while sick-listed then the sickness absence benefits may be paid directly to the individual.

The official retirement age in Denmark is increasing but it was 65 years during the present study. Individuals have the possibility to retire at an earlier age. One such possibility and the most common is the Voluntary Early Retirement scheme. The scheme provides possibility to retire up to five years ahead of the official retirement age. However, for the scheme to become an option, one must have contributed continuously to the scheme for a minimum of 30 years. Individuals may additionally retire early by the use of their own savings or by part or full disability pensioning – accessible to all with severe permanent disability.

C)

Table A. The ELMA and Crude mean results (in days incl. 95% confidence interval) by the expected duration (+/-) of disability pension, pension, and death when compared to the absolute duration time of individuals without depressive and anxiety disorders (reference). Grouped by gender, age, and by disorder: anxiety, depressive, or both.

Gender	Age	Depressive/Anxiety	Disability Pension		Pension		Death	
			ELMA	Crude	ELMA	Crude	ELMA	Crude
			Days (95% CI)	Days	Days (95% CI)	Days	Days (95% CI)	Days
Men	18-47 years	Dep. No/Anx. No (ref.)	0.1 (-80.0:80.2)	0	-	-	0.1 (-140.1:140.4)	0
		Dep. No/ Anx. Yes	+ 112.5 (-0.8:225.8)	-	-	-	- 0.2 (-198.6:198.1)	-
		Dep. Yes/ Anx. No	+ 0.1 (-113.2:113.3)	+ 0.6	-	-	- 23.2 (-221.6:175.2)	+ 0.4
		Dep. Yes/ Anx. Yes	+ 0.6 (-112.7:113.9)	+ 2.2	-	-	+ 48.1 (-150.2:246.5)	-
	48-64 years	Dep. No/Anx. No (ref.)	0.7 (-82.1:83.5)	0.4	27.0 (25.9:28.0)*	27.4	0.3 (0.2:0.5)*	0.5
		Dep. No/ Anx. Yes	+ 0.8 (-116.3:117.9)	+ 0.8	+ 8.5 (7.1:10.0)*	+ 2.1	+ 0.2 (-0.0:0.5)	+ 0.1
		Dep. Yes/ Anx. No	+ 31.0 (-86.1:148.1)	-	- 7.8 (-9.2:-6.3)*	- 8.1	+ 0.2 (-0.1:0.5)	+ 0.2
		Dep. Yes/ Anx. Yes	0.0 (-117.1:117.0)	+ 0.4	- 6.9 (-8.3:-5.4)*	- 3.0	+ 0.5 (0.2:0.7)*	+ 1.9
Women	18-47 years	Dep. No/Anx. No (ref.)	0.1 (-83.7:83.8)	0.1	-	-	0.0 (-138.3:138.3)	0.1
		Dep. No/ Anx. Yes	- 7.5 (-126.0:110.9)	-	-	-	+ 1.7 (-193.9:197.3)	-
		Dep. Yes/ Anx. No	+ 0.2 (-118.3:118.6)	+ 0.5	-	-	+ 6.3 (-189.3:201.9)	-
		Dep. Yes/ Anx. Yes	+ 0.5 (-117.9:119.0)	+ 0.6	-	-	+ 138.5 (-57.1:334.1)	-
	48-64 years	Dep. No/Anx. No (ref.)	0.3 (-81.0:81.5)	0.4	32.2 (31.0:33.4)*	33.7	-21.9 (-187.2:143.4)	0.3
		Dep. No/ Anx. Yes	- 87.8 (-202.7:27.1)	-	- 5.0 (-6.6:-3.3)*	+ 4.4	+ 23.9 (-209.9:257.7)	-
		Dep. Yes/ Anx. No	+ 1.3 (-113.5:116.2)	+ 1.3	- 15.5 (-17.2:-13.8)*	- 9.1	+ 57.7 (-176.1:291.5)	+ 0.4
		Dep. Yes/ Anx. Yes	+ 1.7 (-113.2:116.5)	+ 1.8	- 14.1 (-15.7:-12.4)*	- 9.2	- 31.2 (-265.0:202.6)	+ 0.7

*: 5% significant. Dep. Depressive. Anx.: Anxiety. ELMA: Expected Labor Market Affiliation

Bech, P., N. A. Rasmussen, L. R. Olsen, V. Noerholm, and W. Abildgaard. 2001. 'The sensitivity and specificity of the Major Depression Inventory, using the Present State Examination as the index of diagnostic validity', *J Affect Disord*, 66: 159-64.

Madsen, Per Kongshøj. 2005. 'How can it possibly fly? The paradox of a dynamic labour market in a Scandinavian welfare state', *CARMA; Aalborg University*, 2.

Psychiatric Research Unit
Psychiatric Centre North Zealand, Denmark

Major (ICD-10) Depression Inventory

The following questions ask about how you have been feeling over the last **two weeks**. Please put a tick in the box which is closest to how you have been feeling. A higher number signifies a higher degree of depression.

	How much of the time in the last two weeks…	All the time	Most of the time	Slightly more than half the time	Slightly less than half the time	Some of the time	At no time
1	Have you felt low in spirits or sad?	5 ☐	4 ☐	3 ☐	2 ☐	1 ☐	0 ☐
2	Have you lost interest in your daily activities?	5 ☐	4 ☐	3 ☐	2 ☐	1 ☐	0 ☐
3	Have you felt lacking in energy and strength?	5 ☐	4 ☐	3 ☐	2 ☐	1 ☐	0 ☐
4	Have you felt less self-confident?	5 ☐	4 ☐	3 ☐	2 ☐	1 ☐	0 ☐
5	Have you had a bad conscience or feelings of guilt?	5 ☐	4 ☐	3 ☐	2 ☐	1 ☐	0 ☐
6	Have you felt that life wasn't worth living?	5 ☐	4 ☐	3 ☐	2 ☐	1 ☐	0 ☐
7	Have you had difficulty in concentrating, e.g. when reading the newspaper or watching TV?	5 ☐	4 ☐	3 ☐	2 ☐	1 ☐	0 ☐
8a	Have you felt very restless?	5 ☐	4 ☐	3 ☐	2 ☐	1 ☐	0 ☐
8b	Have you felt subdued or slowed down?	5 ☐	4 ☐	3 ☐	2 ☐	1 ☐	0 ☐
9a	Have you been sleeping too little?	5 ☐	4 ☐	3 ☐	2 ☐	1 ☐	0 ☐
9b	Have you been sleeping too much?	5 ☐	4 ☐	3 ☐	2 ☐	1 ☐	0 ☐
10a	Have you suffered from reduced appetite?	5 ☐	4 ☐	3 ☐	2 ☐	1 ☐	0 ☐
10b	Have you suffered from increased appetite?	5 ☐	4 ☐	3 ☐	2 ☐	1 ☐	0 ☐

Total score ☐☐

Psychiatric Research Unit
Psychiatric Centre North Zealand, Denmark

Depression Inventory MDI: Scoring key

At the top the diagnostic demarcation line is indicated. The total score of the10 items is filled in below

The diagnostic demarcation line

		**How much of the time...**	All the time	Most of the time	**Slightly more than half the time**	Slightly less than half the time	Some of the time	At no time
**Core symptoms**	**1**	**Have you felt low in spirits or sad?**	**5**	**4**	**3**	**2**	**1**	**0**
	**2**	**Have you lost interest in your daily activities?**	**5**	**4**	**3**	**2**	**1**	**0**
	**3**	**Have you felt lacking in energy and strength?**	**5**	**4**	**3**	**2**	**1**	**0**
**Accompanying symptoms**	**4**	**Have you felt less self-confident?**	**5**	**4**	**3**	**2**	**1**	**0**
	**5**	**Have you had a bad conscience or feelings of guilt?**	**5**	**4**	**3**	**2**	**1**	**0**
	**6**	**Have you felt that life wasn't worth living?**	**5**	**4**	**3**	**2**	**1**	**0**
	**7**	**Have you had difficulty in concentrating, e.g. when reading the newspaper or watching TV?**	**5**	**4**	**3**	**2**	**1**	**0**
**Highest score**	**8a**	**Have you felt restless?**	**5**	**4**	**3**	**2**	**1**	**0**
	**8b**	**Have you felt subdued or slowed down?**	**5**	**4**	**3**	**2**	**1**	**0**
**Highest score**	**9a**	**Have you been sleeping too little?**	**5**	**4**	**3**	**2**	**1**	**0**
	**9b**	**Have you been sleeping too much?**	**5**	**4**	**3**	**2**	**1**	**0**
**Highest score**	**10a**	**Have you suffered from reduced appetite?**	**5**	**4**	**3**	**2**	**1**	**0**
	**10b**	**Have you suffered from increased appetite?**	**5**	**4**	**3**	**2**	**1**	**0**

Total score (item 1 – 10) ☐☐

Diagnosis: ICD-10 ____________________ DSM-IV____________________

Psychiatric Research Unit
Psychiatric Centre North Zealand,
Denmark

Major Depression Inventory (MDI): A depression questionnaire with a dual function
MDI: Scoring instructions

The questionnaire consists of the ten symptoms contained in the World Health Organization WHO's depression demarcation. WHO employs the last two weeks as the period of time in which to assess whether each symptom has been present for more than half the time. These symptoms are mainly subjective; therefore it is natural to ask the patient to complete the questionnaire, allowing the patient to tick each symptom. A higher number signifies a more constant presence of the symptom in question. Remember to fill in patient name and the date
The patient's completed questionnaire is scored using the scoring key. MDI (Major Depression Inventory) has a dual function, as it is scored both as an instrument of severity (A) similar to the Hamilton Depression Scale, and (B) as a diagnostic tool.

(A) If MDI is used as a **rating scale** in the same way as the Hamilton scales, then the sum of the ten questions indicates the degree of depression. For items 8, 9 and 10, with two answer categories for each (a) and (b), the highest score is used. The theoretical score range is thus from 0 (no depression) to 50 (maximum depression).

Mild depression:	MDI total score from 21 to 25
Moderate depression:	MDI total score from 26 to 30
Severe depression:	MDI total score of 31 or higher

(B) MDI as a **diagnostic tool**: the vertical line (the diagnostic demarcation line) is used as indicated above. The three top symptoms which reflect the core symptoms of the WHO/ICD-10 diagnosis of depressions must have been present during the last two weeks for most of the time. The accompanying symptoms in the remaining seven MDI items must have been present during the last two weeks for more than half of the time.

The ICD-10 algorithm:

Mild depression:	2 core symptoms and 2 accompanying symptoms
Moderate depression:	2 core symptoms and 4 accompanying symptoms
Severe depression:	3 core symptoms and 5 accompanying symptoms.

MDI can also be employed when diagnosing DSM-IV major depression. According to DSM-IV only nine symptoms are used, as the DSM-IV item 4 is included in item 5. Thus the item with the highest score is used here.

The DSM-IV algorithm:

5 out of the 9 symptoms should be present. Of these one should be one of the two first items; according to DSM-IV these are core symptoms.

A more precise major depression diagnosis depends on the answer to item 9 (a) or (b) and to item 10 (a) or b).

Major depression without inverse neurovegetative symptoms: a score on 9a and 10a.

Major depression with inverse neurovegetative symptoms: a score on 9b and 10b.
